# Engineering poly- and micelleplexes for nucleic acid delivery – A reflection on their endosomal escape

**DOI:** 10.1016/j.jconrel.2022.12.008

**Published:** 2023-01

**Authors:** Benjamin Winkeljann, David C. Keul, Olivia M. Merkel

**Affiliations:** a Department of Pharmacy, Ludwig-Maximilians-University Munich, Butenandtstrasse 5-13, Haus B, 81377 Munich, Germany; b Center for NanoScience (CeNS), Ludwig-Maximilians-University Munich, 80799 Munich, Germany

**Keywords:** Proton sponge hypothesis, Polyethyleneimine, Nonviral vector, RNA, Vaccine

## Abstract

For the longest time, the field of nucleic acid delivery has remained skeptical whether or not polycationic drug carrier systems would ever make it into clinical practice. Yet, with the disclosure of patents on polyethyleneimine-based RNA carriers through leading companies in the field of nucleic acid therapeutics such as BioNTech SE and the progress in clinical studies beyond phase I trials, this aloofness seems to regress. As one of the most striking characteristics of polymer-based vectors, the extraordinary tunability can be both a blessing and a curse. Yet, knowing about the adjustment screws and how they impact the performance of the drug carrier provides the formulation scientist committed to its development with a head start. Here, we equip the reader with a toolbox - a toolbox that should advise and support the developer to conceptualize a cutting-edge poly- or micelleplex system for the delivery of therapeutic nucleic acids; to be specific, to engineer the vector towards maximum endosomal escape performance at minimum toxicity. Therefore, after briefly sketching the boundary conditions of polymeric vector design, we will dive into the topic of endosomal trafficking. We will not only discuss the most recent knowledge of the endo-lysosomal compartment but further depict different hypotheses and mechanisms that facilitate the endosomal escape of polyplex systems. Finally, we will combine the different facets introduced in the previous chapters with the fundamental building blocks of polymer vector design and evaluate the advantages and drawbacks. Throughout the article, a particular focus will be placed on cellular peculiarities, not only as an additional barrier, but also to give inspiration to how such cell-specific traits might be capitalized on.

## Graphical abstract

**Figure:**
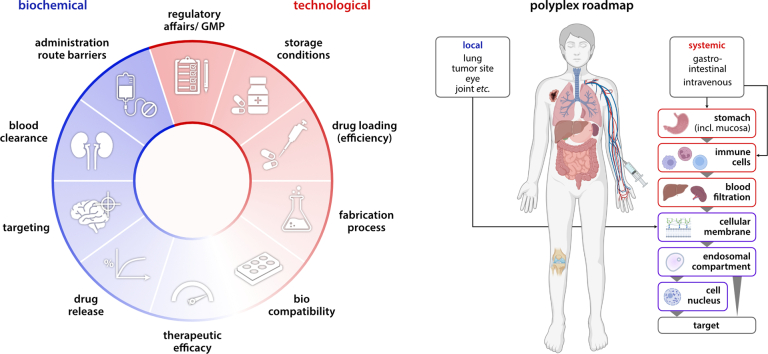

## 1 Introduction

The successful silencing of individual genes in *C.elegans via* RNA interference (RNAi) in 1998 [1] marked a milestone for the future development of RNA-based therapeutics. In 2006, Andrew Fire and Craig Mello were awarded the Nobel Prize in Physiology or Medicine for their discovery. Yet, it was 20 years after their first discovery, in 2018, when the FDA approved *Patisiran* against hereditary ATTR amyloidosis as the first drug to use RNAi in clinical practice [[2], [3], [4]]. Just two years later, boosted by the rapid spread of the SARS-CoV-2 virus, the first mRNA-based vaccine was approved in a record-breaking time [5,6]. As it is indisputable that RNA-based therapeutics are the most promising class of new drugs in the near future, it is also clear that without suitable vector systems, the progress will soon grind to a halt.

Currently, all approved formulations either employ conjugation strategies [[7], [8], [9]] or make use of lipid-based nanoparticles to facilitate trafficking of the RNA therapeutic to its target *loci*. For the latter, PEGylation has become the gold standard to increase the blood circulation time and thus the overall efficiency of the therapy. Unfortunately, PEGylation of nanoparticles can negatively affect both their cellular uptake and endosomal release efficacy. This discrepancy between the benefits and drawbacks of nanoparticle PEGylation is commonly referred to as the “*PEG dilemma*” [10] [11]. Moreover, the exposure of the human immune system to PEG is known to induce the production of anti-PEG antibodies. Consequently, this production of IgM against PEG not only leads to reduced efficacy of the PEG containing therapeutic through the accelerated blood clearance (ABC) phenomenon but can, in some cases, even lead to life-threatening anaphylactic reactions. Furthermore, the antigenicity of PEG can be expected to become increasingly important with the increasing exposure to RNA vaccines and RNAi therapeutics. [[12], [13], [14], [15], [16]]

As an alternative to lipid carriers, polymeric nanoparticles have been researched extensively in the past decades [[17], [18], [19], [20]]. Of course, one must note that the term ‘*polymeric vector’* refers to a multitude of different systems. To be able to cover the topic in a proper depth, we here decided to focus on polymeric systems that encapsulate the nucleic acid cargo *via* either polyelectrolyte complex (short: *polyplex*) or polyplex micelle (*micelleplex*) formation. Although bearing their own drawbacks, such as inherent toxicity in case of too high amine densities or their dynamic equilibrium state, different studies have pinpointed that those polycationic systems offer distinct advantages compared to their lipid-based counterparts [[21], [22], [23]]. In general, both poly- and micelleplexes have been shown to exhibit supreme encapsulation efficiency. Both variants complex nucleic acids by electrostatic interactions as established through protonated amines [24,25]. Whereas even approved lipid formulations such as *Comirnaty* show relatively low drug loading of roughly 4% (w/w) of RNA per lipid nanoparticle (LNP) [26], polyelectrolyte complexes can be formulated with RNA-to excipient ratios of one or more [[27], [28], [29]]. In addition to the electrostatic complexation of nucleic acids, micelleplexes offer the opportunity to co-encapsulate a hydrophobic drug such as P*aclitaxel* [[30], [31], [32], [33]], which has drawn significant attention from oncologists.

The most commonly used and intensively studied polycations in the context of nucleic acid delivery indeed comprise polyethyleneimine (PEI), polyamidoamine (PAMAM), polylysine (PLL), poly(beta-amino ester) (PBAE) or chitosan derivates, but also polymers based on peptides, glycans or spermines are used [[34], [35], [36], [37], [38]]. With the decision for the primary encapsulation agent, further modifications of the system are possible. Those not only refer to whether or not to introduce hydrophobic moieties but may also include the incorporation of targeting ligands or membrane penetrating peptides [[39], [40], [41], [42], [43]].

**Fig. 1:**
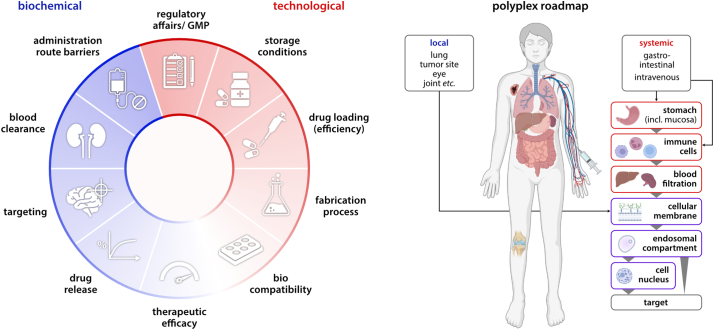
Boundary conditions in nanoparticle formulation. Biological and chemical considerations are the daily work of formulations scientists. Detailed knowledge of human physiology is imperative to addressing objectives such as blood clearance, targeting strategies, or therapeutic efficacy. However, clinical stages can only be reached if technological aspects - including fabrication methods, storage conditions, and GMP regulations - are attributed with equal importance.

However, this sheer limitless toolbox of possibilities concerning carrier polymers and compositions can be both a blessing and a curse; for those who aim at developing a new polyplex formulation, it is a prerequisite to consider a plethora of aspects and boundary conditions that will determine the success of their product. We here clustered those reflections into two main categories, *i.e.*, biochemical and technological considerations, while neglecting the financial aspect an enterprise would additionally have to acknowledge (Fig. 1).

Similar to the development of other nano pharmaceutics, addressing the biological facets regarding the formulation of a polymeric carrier system requires meticulous knowledge not only of human physiology. Moreover, it is crucial to recognize also the pharmacokinetics, *i.e.*, rate and extent of absorption, distribution, metabolism, and excretion (ADME), of the nanoparticular delivery system intended to be used. Therefore, the first considerations comprise the identification of the pharmacological target and an in-depth analysis of the biological route, starting from the desired point of administration to the site of action (Fig. 1). If systemic application is chosen over local administration, avoiding premature blood clearance is imperative for the nanoparticle formulation to even have a chance of reaching its target at a therapeutically sufficient concentration. Substantial progress has been made in the last decades to maximize the blood circulation of polymeric nanoparticles. Unfortunately, the majority of strategies still rely on PEGylation [[44], [45], [46], [47], [48]], which comes along with the problem of ABC phenomena, similar as discussed for LNP formulations.

However, the immune system is just one among a series of biological barriers on the road to success (Fig. 1). After arriving at the target tissue, the nucleic acid-containing nanoparticles must finally cross the extracellular matrix to be taken up by the cells. This step depends not only on the physicochemical properties of the formulation itself but also on the type of target cell both *in vitro* and even more so *in vivo*; whereas the adherent cell line HEK-293, for example, is relatively easy to transfect with plasmid DNA (pDNA), the Jurkat suspension Lymphoma/Leukemia cell line, in contrast, remains a “hard-to-transfect” cell line. [49]

Then, even if the cellular uptake was accomplished, the nanoparticles face another destiny: the endo-lysosomal pathway [50]. Again, time is running against the formulation, as if not being able to escape the endosomal compartment efficiently, the degrading environment of the lysosome will eradicate all chances of the nanomedicine to become therapeutically active. Different studies have assessed the endosomal escape of polymeric systems and found that only a few percent of the nanoparticles that accomplished being taken up by the target cell are, in the end, able to escape the endosome [[51], [52], [53]]. Yet, even if all boundary conditions have been met perfectly, an efficient therapy could still fail due to insufficient release of the therapeutic nucleic acid from the carrier matrix. Consequently, the accumulation of unproductive and non-degradable material, such as PEI in the cytoplasm, will interfere with the metabolism of the cell and potentially lead to cell death. [54] Notably, the question of long-term biocompatibility and excretion of non-biodegradable components remains not fully understood. [20]

With the focus turned on targeting, it is crucial also to consider the fate of missed targets. Investigating the biodistribution of the formulation *in vivo* is the first decisive step to assessing the extent of off-target accumulation and evaluating the expected intensity of attainable side effects. Depending on the target site, the situation can differ quite drastically; whereas targeting organs such as the liver or spleen naturally results in a favorable ratio of on- to off-target drug accumulation, aiming at other organs, particularly in the central nervous system, will result in dramatically lower targeting efficiencies if no specific drug carrier design was implemented. [55] If possible, tuning the system to achieve selective targeting may be a strategy to minimize the repercussions of side effects. [56] Nevertheless, ligand-mediated targeting is still far from reaching perfection; Chan and colleagues demonstrated a few years ago that less than 14 out of 1 million gold and silica nanoparticles particles with a specific targeting ligand reached the designated target cells [57]. How those data transfer to polyplex systems is yet to be shown.

At first glance, the second half of the puzzle – the technological aspects – does not seem to be of utmost importance for academic research. Yet, without considering their influence, it is likely that even a pharmacologically successful formulation is going to fail the step into the clinic. In analogy to the assessment of the biological route, all steps of the fabrication process require attention on an individual basis (Fig. 1). Most importantly, reproducibility of the formulation must be achieved, including the composition and purity of synthesis products and the structural and physical characteristics of the formulated nanoparticles [58]. It is indisputable that the inability to scale up the production to human-scale would manifest a K.O. criterion when it comes to commercialization of the formulation [38]. Additionally, both loading and loading efficiency come into play: whereas the first, as it changes the drug to excipient ratio, has a significant impact on the biocompatibility of the whole formulation, the latter is decisive to avoid that the usually expensive nucleic acid therapeutic is unnecessarily wasted. It might not be paramount for the phase of clinical trials, but at the latest, when a product is launched into the market also, the conditions required for storage of the drug formulation become pressingly important. Although the COVID-19 pandemic has impressively demonstrated that highly complex low-temperature infrastructures are feasible, this technology, in the long run, will remain exclusive for rich countries and states in regions of cold to moderate climate [59,60]. Technological strategies to overcome the necessity of low-temperature storage of RNA therapeutics are primarily based on freeze-drying [61], yet room temperature stable formulations have also been generated *via* spray drying [[62], [63], [64]]. Furthermore, significant boundary conditions for the technological processes are dictated by the regulatory instances of the corresponding markets, *e.g.*, the FDA or EMA. Especially for the pharmaceutical industry, those regulations and good manufacturer practices (GMPs) are typically orders of magnitude more detailed when compared to other industrial sectors; thus, an early assessment will later become advantageous and provides an opportunity for a head-start.

Even though centering our focus on polymeric nanoparticles, thoroughly discussing all prospects and biological fundamentals influencing rational engineering can become exhaustive. In this review, we thus decided to spotlight the endosomal pathway of poly- and micelleplexes and draft design guidelines around this focal point. Therefore, we will start with an overview of the endosomal compartment in eukaryotic cells while highlighting cell-specific variations along this pathway. In the following, we will review both the most important and recent studies on endosomal escape mechanisms for polycationic drug carriers. Again, we will include cell-specific considerations and point out gaps that have not been sufficiently addressed yet. Based on this analysis of the intracellular trafficking, we will draw conclusions and transcribe those ideas into a toolbox to help the reader design a *de-novo* polymeric nanoparticle formulation.

## 2 Intracellular trafficking of polymeric drug delivery vectors

As introduced above, the escape from the endosomal compartment represents one of the major bottlenecks that limit the successful delivery of nucleic acid therapeutics. To allow for a design of nanoparticle systems capable of overcoming this barrier efficiently, one should be aware of the biological peculiarities of endosomal trafficking. The exact mechanisms have been reviewed thoroughly elsewhere [65]. Yet, to be later able to conclude the design polyplex systems, we will concisely depict the possible pathways a drug carrier might take, starting from the cellular uptake and ending up in either degradation or recycling.

### 2.1 General information on the endosomal-lysosomal compartment

In general, endocytosis is referred to as the internalization of fluid, solutes, macromolecules, plasma membrane components, or of drug delivery vehicles. The process is achieved by the *de novo* production of intracellular membranes from the plasma membrane and, therefore, the formation of intracellular vesicles [66,67]. The two main types of endocytosis are generally classified according to the materials to be internalized; fluid droplets and solubilized substances are usually taken up *via* pinocytosis, whereas solid, particular substances are internalized through phagocytosis. A further sub-classification of pinocytosis into clathrin-mediated, macropinocytosis, caveolin-mediated, clathrin- and caveolin-independent pathways involves the molecular contributors of the vesicle formation [68].

**Fig. 2:**
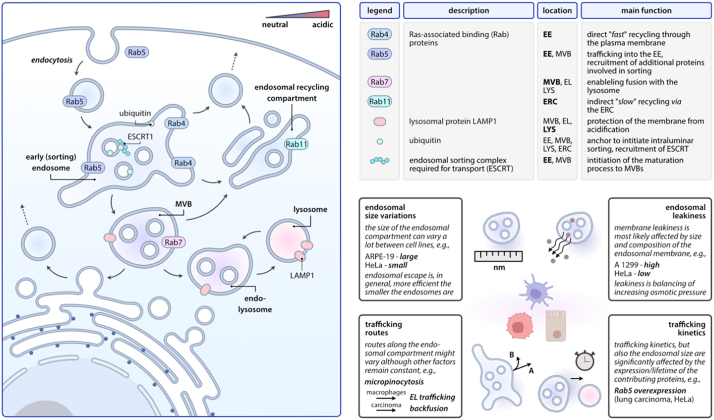
Biological route and cell-specific details. Cargos taken up *via* endocytosis are trafficked through the endosomal compartment, where they are predominantly either degraded or recycled. Multiple markers along the pathway determine the exact trafficking route. Although the general structure of this pathway is similar in all cells, specific alterations are found throughout different cell types.

After endocytosis, the cargo, in general, is processed within the endosomal-lysosomal system. The endosomal compartment thereby operates as a central sorting unit of the cell. It differentiates those cargos intended to be recycled or exocytosed through back fusion with the plasma membrane and those intended to be further processed within the lysosomal system. Here, the first step comprises the processing of the cargo in the early endosomal (EE), which is thus also referred to as “sorting” endosome (Fig. 2). [69]

The process of internalization, as well as maturation into the EE, is consistently driven by a group of proteins with GTPase activity called Ras-associated binding (Rab) proteins and Rab effectors. These groups of proteins are involved in processes such as vesicle tethering, fusion, budding, and motility [69]. Rab proteins associated with the EE predominantly include Rab5 and Rab4 [70]. Here, Rab5 plays an essential role in regulating the trafficking of cargo to the EE, homotypic fusion, motility, and exhibits additional functions in the signaling pathway. Rab5 can further recruit Rab effector proteins, such as early endosomal antigen-1, PtdIns(3)P-kinase/Vps34, Rabenosyn-5, APPL1, and APPL2 involved in the endosomal sorting process and homotypic fusion of EEs. The recruitment of PtdIns(3)P-kinase/Vps34 leads to a phosphorylation of the lipid phosphatidylinositol (PtdIns) which then can exclusively be found on the surface of the EEs. PtdIns(3)P can in turn bind additional Rab effector proteins, such as hepatocyte growth factor-regulated tyrosine kinase substrate (Hrs), which will be described later. [67,69].

Individual EEs may differ from each other in terms of morphology, localization, composition, and function. Furthermore, their distribution can vary quite drastically dependent on the cell type [67]. Components, intended to be recycled accumulate within the membrane of the approximately 60 nm diameter large tubular extensions of the EE. Proteins intended for further endo-lysosomal processing are preferably concentrated in the lumen of the intraluminal vesicles (ILVs) which are approximately 400 nm in diameter [69]. This spatial sorting within the EE is accompanied by a pH gradient ranging from pH ∼6.5 in the periphery to pH 6.2-5.5 in the central region and ILVs. The membrane of the vacuolar domains typically shows an increased content of cholesterol when compared to the tubular regions. Furthermore, a large amount of sphingolipid-rich rafts, membrane protein aggregates, V-ATPases, clathrin, “endosomal sorting complex required for transport” (ESCRT1), and membrane proteins destined for degradation is associated with the ILVs. [67,69]

Whereas sorting for recycling is mainly driven by the accumulation geometry achieved by localization of recyclable proteins in the tubular area of the EE, vesicle formation *via* GTPase Rab11/Rab4, and the degradation pathway is primarily controlled by ubiquitination. [69,71]

Molecules intended to be recycled, such as plasma-membrane components or receptor-associated ligands, are restored after Rab4/Rab11 vesicle formation through the endosomal recycling compartment (ERC). This transportation is accomplished by the hydrolysis of PtdIns(3)P and the activation of small GTPase Rab11, which can be found on the surface of the ERC. [72] Additionally, a Rab4-mediated fast-recycling process has been reported, which directly traffics the cargo to the plasma membrane without the involvement of the ERC. [69] This faster-recycling route, measured using recycling rates of the transferrin receptor in human carcinoma A431 cells, was characterized with a t_1/2_ of 7.5 min, whereas the slower route through the ERC was significantly slower (t_1/2_ ≈15-30 min). [73]

Sorting for degradation, in turn, requires ubiquitination. Ubiquitination of lysin residues is recognized by the ubiquitination interacting motif of the hepatocyte growth factor-regulated tyrosine kinase substrate (Hrs). Additionally, Hrs can associate with clathrin in clathrin lattices on EE membranes intended to drive intraluminal vesicles away from the tubule-forming area. Finally, Hrs interacts with the Tsg101 subunit of ESCRT1, leading to the formation of multivesicular bodies (MVBs) and, therefore, to the maturation into late endosomes (LEs). [67,69]

During the maturation of the EE to LE, Rab5 is exchanged with Rab7, which is responsible for enabling the fusion with lysosomes [74]. The newly formed LEs are typically in the size of 250-1000 nm. [69] Whereas their lumen contains hydrolases, lysosomal proteins, such as LAMP-1, responsible for protecting the membrane during acidification, can be found on the surface. Depending on the cell type, LEs contain acidic multilamellar or multi-vesicular/multilamellar regions with a pH ranging from 6.0 to 4.9. Important additional maturation steps include the conversation of PtdIns(3)P to PtdIns(3,5)P(2), a movement into the perinuclear area, exchange of membrane components, a shift in choice of fusion partners, and a change in morphology. Differences can further be found in the membrane composition within the LEs. Interestingly, the luminal membrane exhibits a detectable level of lysobisphoshatidic acid (LBPA) which can primarily be found in the LEs and which is responsible for the recruitment of hydrolases through charge [67,[75], [76], [77]].

LEs, can fuse with vesicles from the trans-Golgi network (TGN), mediating the transport of lysosomal components, heterotopically with lysosomes and homotopically with themselves. In general, lysosomes are a heterogeneous population of vacuoles in terms of composition, morphology, location, and density. Those fusion and fission processes observed between LEs and lysosomes are associated with exchange of membrane components as well with cargo and protein transfer. The fusion of LE and lysosomes is typically referred to as intermediate endo-lysosome (EL). [75]. Of note, a clear differentiation between LE and lysosome is not always possible as all involved proteins can be found on either of their surfaces.

### 2.2 Examples of intercellular differences

As mentioned above, the endosomal fraction can vary both within an individual cell and, of course, between different cell types [67]. We will discuss the differences in the cellular uptake pathway within different cell types in the following. Our discussion will illustrate rather exemplarily consequences of such endosomal differences then giving an exhaustive summary also while many details remain to be investigated.

Macropinocytosis, which typically occurs in highly ruffled regions, for example in macrophages (*e.g.,* HEK293, COS-1) and brain microvascular endothelial cells, leads the cargo to be trafficked similarly to the EE pathway as described above. In contrast, endocytosis via micropinocytosis in certain carcinoma cell lines (*e.g.*, A531) primarily results in a back fusion with the plasma membrane and recycling of the cargo. Similarly, different trafficking after caveolae-mediated uptake can, for example, be observed between endothelial cells and non-endothelial cells such as smooth muscles, fibroblasts, and adipocytes. Whereas transcytosis is the primary pathway after caveolae-mediated uptake, the latter traffics the cargo into the EE. [66,78,79]

Furthermore, variations in the endosomal pH were found depending on the cellular function; dendritic cells (DCs), as professional antigen-presenting cells, are capable of what is commonly referred to as cross-presentation. Cross-presentation, in brief, describes the process of presenting an extracellular antigen on major histocompatibility complexes (MHC), *e.g.,* MHC Type I to CD8+ T-cells [80]. To avoid protein degradation, DCs can increase the pH value in the phagosome (which is the equivalent of the endosome after internalization *via* phagocytosis). This increase in the pH is achieved by recruiting NADPH oxidase (NOX II) to the early phagosome. The permanent production of reactive oxygen species then leads to alkalization of the phagosome. NOX II-mediated alkalization was not observed in macrophages, however, which are primarily part of the innate immune reaction for the elimination of microorganisms [81].

Moreover, depending on the maturation status of DCs, the endosomal sorting can potentially change; in immature DCs, endocytosis and sorting of MHC II molecules into ILVs requires ubiquitylation for lysosomal degradation. Maturation of DCs leads, however, to decreased ubiquitylation. In a maturation state, MHC class II molecules are still initially trafficked into ILBs, yet, they were later found to be exocytosed through the plasma membrane [82]. A recent study published by Shearer *et al.* on the appearance of endosomal maturation markers further highlighted these variations between different cell types or phenotypes. There, the authors chose Rab5 as EE marker, Rab7 as LE marker, and LAMP-1 as lysosomal marker to evaluate the endosomal pathway *via* confocal microscopy. They observed colocalization of Rab5 and LAMP-1 in C2C19 myoblast cells and A549 human adenocarcinoma cells indicating a fusion of the EE with lysosomes before maturation. The degree of colocalization was more pronounced in A549 cells, highlighting the subtle differences that can exist in endosomal maturation processes amongst different cell lines. Although colocalization of all chosen markers was observed in both cell lines, significant differences in their amount were detected. The authors hypothesized a higher possibility of EE and lysosome fusion events in A549 cells potentially influencing liposome recycling, [83] which could possibly lead to reduced receptor recycling.

## 3 Endosomal escape mechanisms of polymeric nanocomplexes

**Fig. 3:**
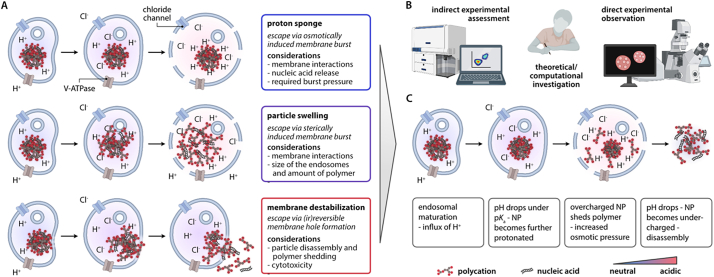
Escape strategies and how to assess them. Different individual mechanisms have been proposed for how polyplexes can escape the endosomal compartment (A). Both experimental and theoretical investigations continue to shade light onto the dark (B). The newest findings suggest that, depending on the particular delivery system, combinations of those postulates are more likely to reflect the reality than individual processes (C).

After a detailed look at the current literature about the endosomal compartment and some of its biological and cell-specific peculiarities, the most important question for a drug nano formulation is how to escape this pathway to reach the cytosol. For lipid-based formulations, many details regarding their endosomal pathway have been unveiled; for example, most researchers agree that it is of utmost importance for LNPs to escape the endosomal compartment before reaching the degradation pathway, *i.e.*, in the sorting endosome or within one of the different recycling pathways (see Chapter 2 for details). Studies investigating the endosomal escape of polymeric NPs are significantly less frequent than those focusing on LNPs. Nevertheless, different theories have been postulated, discussed, and refined over the years. The most influential ones include the proton sponge theory, as well as particle swelling and membrane destabilization (Fig. 3A) [68,84,85].

### 3.1 Different theories and their origin

The term “*proton sponge*“ was initially coined to refer to a group of aromatic diamines with exceptionally high basicity; however, more commonly, it is used to describe the escape of polyamine-based nanoparticles from the endosomal pathway by causing them to burst. The proton sponge theory was first described by the chemist Jean-Paul Behr in the 1990s [86], along with an increasing interest in polycations such as polyethyleneimine for the application as non-viral gene delivery vectors [87]. The proton sponge effect claims that the uptake of polycationic structures with a buffering capacity will induce an increased osmotic pressure within the maturing endosome which ultimately leads to its burst. In particular, Behr argued that polyethyleneimine becomes further protonated upon acidification along the endosomal pathway. As this protonation prevents the endosome from lowering its pH value, ion pumps encounter this buffering effect by sustaining the proton influx into the lumen of the endosome. At the same time, a passive influx of chloride ions becomes necessary to maintain the charge equilibrium. Subsequently, this osmotic gradient leads to water uptake resulting in an increased osmotic pressure until, upon a certain intracompartmental pressure, the endosomal membrane collapses (Fig. 3A).

In contrast to the proton sponge theory, which makes the optimistic assumption that there is no interaction between polyplex and endosomal membrane, other studies consider this interface. Literature usually differentiates between hole formation as induced by an intact polyplex and hole formation through intercalations of free polymer with the endosomal membrane (Fig. 3A). Especially the first adds another dimension to the whole process, as it challenges the question of how and when the carried load should be released if the polyplex were to leave the endosome as a whole.

### 3.2 Tracking down the endosomal escape

Even today, these endosomal escape mechanisms are discussed controversially. About four years ago, Vermeulen *et al.* presented a short review discussing several studies providing evidence against or favoring the proton sponge hypothesis [84], and Bus *et al.* summarized alternative endosomal mechanisms through increased membrane permeability [85]. Complementary review articles further discussed different endosomal escape mechanisms - not always focusing on polyplex systems solely but often including other non-viral gene delivery vectors [[88], [89], [90], [91], [92]]. In the last few years, additionally, several high-quality studies have been published, shedding new light onto the topic. To structure the different investigations in a comprehensive manner we decided to cluster them in either theoretical/computational, indirect, and direct experimental approaches (Fig. 3B).

#### 3.2.1 Indirect experimental assessment of endosomal escape

Indirect experimental approaches are historically the first attempts to investigate endosomal escape as they are the simplest approach to test a postulated theory; through the variation of different in- and output parameters, the theory can be either strengthened, forced to be refined, or proven wrong. Regarding the proton sponge theory, indirect experimental assessment initially focused mainly on the input parameter of buffering capacity and output of transfection efficacy. Additionally, methods were employed to inhibit the activity of proton pumps, *e.g.*, by treating the cells with Bafilomycin A1 prior to transfection. The most frequently employed model systems comprise PEI and PLL since they exhibit a comparable net charge density while exhibiting a high (PEI) versus almost no buffering capacity (PLL) around the endosomal pH level. Consistently, correlations were found if either the buffering capacity of PEI or PLL were altered by chemical modification or the buffering mechanism of the endo-lysosomal compartment was modified [[93], [94], [95]]. Similarly, studies were conducted observing trends that might contradict the proton sponge theory. However, as discussed before, especially chemical polymer modifications might influence additional parameters, including cellular uptake (pathways), carrier size, zeta-potential and loading, or release/dissociation of the complex.

Along with both increasing interest in polycationic drug carriers and the development of new experimental techniques, interest in solving the riddle rose again, and the number of unsolved phenomena was systematically lowered. In 2013 Benjaminsen *et al.* employed a nanoscopic pH sensor to assess the acidification within the endosomal compartment upon transfection with PEI. The authors have shown that the endocytosis of PEI-based polyplexes does not prevent the endosome/lysosome from acidification; however, the authors added that this does not exclude the possibility of the osmotic pressure being increased through the influx of hydrogen and chloride ions. Additionally, the authors used the Yong-Laplace relationship to estimate the critical radius the lysosome would have to reach to burst, with the outcome that this event seems unlikely. It was concluded that even though possible, the proton sponge-induced rupture is unlikely to be the dominant effect that influences the transfection efficacy. [96,97]

In contrast, a very recent study by Roy *et al.* indeed did observe a buffering capacity of PEI that was able to reduce the acidification along the endo-lysosomal pathway. It was discussed that the absence of the buffering effect, as observed by Benjaminsen *et al.,* might be caused by the lack of colocalization between the sensor and PEI. Indeed, Benjaminsen *et al.* indirectly assessed the colocalization by showing that both PEI and lysosomes, as well as lysosomes and pH sensors colocalize; Roy *et al.*, however, investigated with a direct method. [97,98] Nevertheless, the latter study exclusively focused on pH buffering. An investigation on the actual endosomal escape was not conducted, thus not answering the question whether the endosomal pressure generated by the buffering PEI would be sufficient to induce an endosomal burst.

The problem with interpreting colocalization or its absence is frequently found when using fluorescence-based methods. Beach et al. recently proposed a novel technique to overcome these limitations in the context of endosomal escape. This technique, which the authors refer to as the “*split luciferase endosomal escape quantification*” assay, makes use of two components, a protein (LgBiT), which is engineered to be expressed by the cell, and a complementary peptide (HiBiT), which is conjugated to the drug carrier. A luminescent signal is expressed if those two are in close proximity, i.e., when the NP reaches the cytosol. As luminescence is the result of HiBiT from the nanoparticle core combining with cytosolic LgBiT, any signal measured directly corresponds to the endosomal escape of the nanoparticle. Using this methodology, the authors showed that those NPs that disassemble at an earlier stage of the endosomal compartment show a better endosomal escape than those disassembling at very acidic pH [99].

Consequently, also the questions arose about which role interactions between cationic polymers and the endosomal membrane play when it comes to endosomal escape. Vaidyanathan *et al.* studied the interaction of PEI, PAMAM, and their respective polyplexes with the cell plasma membrane. As a measure of intercalation, the change in membrane conductivity was assessed. Indeed, the data suggest that those polymers that intercalate into the membrane can form pores and remain there for extended times, thus possibly offering pathways for the endosomal escape of polyplexes [100]. In their follow-up study, the authors further investigated the transfection efficiency of PEI and PAMAM polyplexes, again highlighting that intercalation of free polycations with the endosomal membranes plays a vital role in endosomal escape. The question remains why PEI showed significantly better transfection efficiency as the - according to the authors’ hypothesis - required difference in the efficiency of membrane intercalations between the two polymers has yet to be investigated [101]. However, differences in membrane interactions have also been addressed by other studies. Although conducted with protein-loaded polyplexes, the findings by Kretzmann *et al.* suggest that also polymer geometry plays an important role when it comes to membrane intercalation; tracking their polyplexes along with the cellular compartments *via* confocal microscopy and assessing their transfection efficacy, the authors concluded that large multivalent, flexible structures tend to interact stronger with plasma membranes when compared to rigid (spherical) geometries [102]. Further support for the importance of membrane interactions is found by Rehman *et al.,* who used spinning disk microscopy to track the route of their polyplexes in living cells. Also, here, the data suggest the formation of pores rather than a destructive endosomal burst, thus, further highlighting that membrane interactions must not be ignored in this context [52]. The authors additionally evaluated the number of particles that are actually involved in the transfection of a cell. To do so, the cellular uptake of two types of polyplexes containing either red or green fluorescently labeled oligonucleotides (ODN) was evaluated in HeLa cells. Interestingly, only 23% of nuclei of the HeLa population were positive for both ODNs, whereas the majority of 77% showed either only red or green fluorescence after transfection. If the carrier in contrast was formulated using red and green labeled ODNs or plasmids, all nuclei exhibited a yellow signal of colocalization. Based on those findings Rehman *et al.* concluded that the delivery of as few as 1-5 polyplexes per cell could be sufficient for a successful transfection [52].

Additional support for the importance of membrane interactions can be found in a study by Clark *et al.* [103]. There, the authors investigated the effects of PEI adsorption on the properties of dipalmitoylphosphatidylcholine (DPPC) and bis(monoacylglycerol)phosphate (BMP) model vesicles by confocal microscopy and dynamic light scattering. Controversially, their results suggest that PEI adsorption increases the overall integrity of DPPC/BMP vesicles, thus rendering them more resistant to osmotic pressure. However, PEI adsorption was observed to mediate deformation and permeabilization of the vesicle, allowing even large model cargos (> 1 kDa) to diffuse out.

A very recent study took systematic investigations to a new level; Cho *et al.* presented a chemically tunable core-shell NP system to meticulously decipher the chemical structure-transfection efficiency relationship of polymeric vectors. Cellular uptake and knockdown were assessed *via* flow cytometry, and confocal microscopy was added to visualize endosomal escape. The authors investigated the efficiency in relation to proton buffering, acid degradability and membrane disruption ability. Interestingly, comparable transfection efficiencies were observed even if the buffering capacity of the used core-shell nanoparticle was substantially increased. Those findings indicate that the proton sponge mechanism on its own may not be sufficient to achieve endosomal escape of the NPs. Despite the authors considering acid degradability as more important, improving both buffering capacity and acid degradability simultaneously lead to significantly enhanced transfection efficiency. Of note, the authors themselves mention a few differences based on the monomers used, *e.g.*, the different zeta potential of the NPs, which ideally would need to be addressed before the results can be interpreted without any remaining doubt[104]. These differences in the physical properties of NPs that go along with chemical alteration might, in fact, be the most limiting factor in such systematic but indirect investigations; the alignment of all relevant parameters affecting the endosomal escape while only investigating one alteration at a time appears to be impossible.

#### 3.2.2 Theoretical and computational investigations on the endosomal escape of polycationic complexes

In addition to systematic parameter variations to indirectly assess endosomal escape mechanisms, early theoretical attempts and models aimed at estimating whether or not the endosomal burst as proposed in the proton sponge hypothesis is actually feasible.

By assuming a model polyplex composed of five plasmid DNAs and polyethyleneimine, Won *et al.* estimated that the resulting strain on the endosomal membrane upon a pH drop from 7.4 to 5.0 would reach 2.3 % [105]. Comparing this value with burst strains of lipid vesicles, which are typically in the order of 2 to 5 %, at least suggests that an endosomal burst could be possible [106]. The estimation the authors made of the polyplex composition therefore also indirectly pinpoints that the number of complexed NAs within a single endosome might be crucial. It can be speculated whether a high number of complexed NAs can be more efficiently accumulated within the endosomal compartment via multiple small complexes or individual complexes of larger dimensions.

Already in 2008, Yang *et al.* suggested that an endosomal burst upon a pH drop might be feasible. Their initial model indicated that the pH drop from the cytosol (degree of PEI protonation *p* = 15 %) to late endosome (*p* = 45 %) leads to a compaction of the polyplex and, consequently, a decrease in the osmotic pressure [107]. At that time, this finding was supported by an experimental study investigating the hydrodynamic radii of different polyplexes under varying pH conditions where it was found that, above a certain polymer-to-nucleotide ratio, the particle size decreases with the pH becoming increasingly acidic [108]. In the second step, however, the authors added that a critical value can just be reached if a sufficient amount of free polymer is also present within the endosome. According to their model, an exchange of free and complexed polymer was expected to be unlikely [107].

The ability of uncomplexed polyamines with p*K*_a_ values in a suitable range to enhance osmotic pressure capable of bursting the endosomal membrane was also described by Freeman *et al.* [109]. To prove their hypothesis, the authors proposed and validated an endosomal model including ion channels, pumps, diffusive processes, and an elastic membrane [110], which in their follow-up study was used to investigate the effectiveness of different proton sponges in terms of inducing osmotic pressure on the vesicle membrane. Freeman tested different polymers, including PAMAM, PEI, and PLL, and evaluated that the ideal span for the polymer’s p*K*_a_ value should be between 7.25 and 6.25.

At the same time, different studies reassessed the behavior of polyplexes formed by titratable polymers upon acidification. Using molecular dynamics (MD) simulations, Amoruso *et al.* visualized that a pH drop leads to polymer release from the polyplex, whereas the remaining polymer further compacts with the nucleic acid payload [111]. Similarly, also experimental studies confirmed the shedding of polymers from the polyplex when the pH level drops [112]. In 2021, Bryn Monnery combined the findings of the latter studies to present a modified proton sponge hypothesis, where he explains the endosomal escape of polyplexes by hypothesizing that the released free polymers then disrupt the endosomal membrane *via* acid-catalyzed hydrolysis of phosphate ester bonds [113]. Yet, with the additional knowledge that polyplexes tend to release polymers from the complexed structure along the endosomal pathway, the initial model presented by Yang *et al.* becomes similarly likely, even without the limitation of depending on the additional internalization of free cations together with the polyplex (Fig. 3C).

Yet, it is essential to be reminded that the shedding of polymers from the polyplex might also depend on the physio-chemical properties of the polyplex. In particular, Amoruso and colleagues showed in the same study mentioned above that a polyplex formed by a medium-sized model DNA (100 monomer units) and a similarly long PEI molecule upon a pH drop, although changing its conformation, stays in a complexed state. In contrast, using several short PEI molecules for polyplex formation results in polymer shedding under otherwise similar conditions [111]. From this point, one can recognize that again a matrix of nucleic acid-polymer combinations opens up where each of these combinations can potentially behave differently regarding complexation, shedding, and decomplexation of the respective polyplex.

To be able to cope with that number of possibilities and amount of data, combining MD simulations with machine learning (ML) has emerged as a promising approach [114]. Yet, having a more detailed look at MD simulations reveals another problem; indeed, most polyplex systems are too large, and the timescales are too long to be assessed as a whole *via* classical all-atom MD simulations. Fortunately, the quality of coarse grained (CG) models is also increasing. Using the well-known Martini forcefield, Bruininks *et al.* [115] were able to simulate an entire lipoplex (radius of gyration up to 30 nm) formed by a mixture of DOTAP and DOPE with dsDNA. Moreover, they not only focused on the lipoplex *per se* but investigated its uptake through an endosomal model membrane. Their simulation demonstrated that membrane fusion of the lipoplex with the lipid-bilayer of the endosomal membrane and, therefore, the transfection efficacy critically depends on the membranes’ lipid composition. *Vice versa,* it is likely that also variations in the lipoplex composition will have a significant impact on the fusion with the endosomal membrane.

While the Martini model was initially developed to investigate lipid structures and polypeptides only, it received a significant upgrade in 2021, which offers a toolbox of additional options [116]; based on this upgrade, considerable effort was placed into the parametrization and optimization of CG models for polymeric structures and new tools became available to study these structures in depth [117]. As those steps have not broadly reached the field of drug delivery yet, it appears likely that one can expect major steps in this interdisciplinary field in the near and mid future.

#### 3.2.3 Experimental methods to directly visualize endosomal escape mechanisms

As human perception is in general centered around ocular vision, it is the instinct of many scientists to aim at directly visualizing phenomena rather than theoretically predicting them or drawing conclusions from a systematic variation of indirectly related in- and output data. The Abbé diffraction limit naturally sets a boundary preventing light microscopy from being able to visualize endosomal escape. That is why we decided to count confocal fluorescence microscopy to the indirect experimental techniques for the purpose of this article as the diffraction-based resolution limit, with, even in an ideal case, being around a few hundred nanometers, does not allow for spatially distinguishing events in the size range of individual polyplexes. However, two families of microscopic techniques, i.e., electron microscopy (EM) and super-resolution microscopy (SRM), can be used to circumvent the diffraction limit. Recently Andrian *et al.* presented a detailed review on the use of these techniques to quantify endosomal escape events [118], which is why we will restrict our discussions to a concise overview, with its focus - in line with the structure of this review - set on polyplex systems.

With a near-atomic resolution level, EM is naturally suitable for visualizing events at a subcellular and even molecular level. Consequently, especially transmission electron microscopy (TEM) has been used to capture the endosomal escape of nanoparticles in an image directly. [[119], [120], [121]] Thereby, many studies also tend to investigate the intracellular trafficking of polyplexes employing TEM. [122] Bieber *et al.*, for example, used different fluorescence techniques in combination with TEM to study the intracellular route of PEI-DNA polyplexes. Their high-resolution TEM images clearly depicted the localization of several individual polyplexes per lysosome. Furthermore, the proximity of some of these polyplexes to the lysosomal membrane led the authors to hypothesize about membrane hole formations [123].

Unfortunately, EM techniques bear the inherit problem of requiring a time-consuming sample preparation prior to imaging. In the end, a fixed and sectioned system is observed, and it is hard to predict whether or not the sample after the preparation still reflects the physiological state. Compared to the sample preparation required for SEM and standard TEM, cryo-techniques are certainly less damaging. However, although employed to study the morphology of poly- and micelleplexes [[125], [126], [127], [128], [129]], cryo-EM up to know has only sparsely been used as a visualization technique to study endosomal trafficking and escape of nanomedicines in general [[124], [125], [126]]. To the best of our knowledge, cryo-EM has not been applied to study intracellular trafficking of neither lipid nor polymeric nanoparticles.

Fortunately, with the development of the Nobel prize-winning super-resolution fluorescent techniques PALM (PhotoActivated Localization Microscopy) [127] and STORM (STochastic Optical Reconstruction Microscopy) [128] in 2006, the path was paved to circumvent the diffraction limit of fluorescence microscopy. Further developments in the field of SRM and single-molecule techniques today allow image acquisition speeds fast enough to perform “*quasi*” real-time imaging of the sample [129,130].

Several studies demonstrated that SRM microscopy is a suitable tool for studying poly- and micelleplexes regarding different aspects, e.g., complexation and decomplexation, aggregation, and stability in different environments [[131], [132], [133]]. Equipped with the STORM technique, Wojnilowicz *et al.* in 2019 published a striking study about endosomal burst as described in the original proton sponge hypothesis. In detail, the authors investigated the intracellular route of glycogen/siRNA polyplexes; by additionally labeling the colocalization of individual glycoplexes with endosomes, their escape was nicely observable. Notably, it became clear that these glycoplexes even escaped the endosome as a whole. The authors additionally employed Bafilomycin A1 to inhibit endosomal acidification. This treatment almost completely inhibited the endosomal escape of the glycoplexes, proving that, at least for their particular polyplex system, the buffering effect of the polycations is the major driver for escape from the endosomal compartment. [134] Although focusing on lipid nanoparticles rather than polyplexes, Paramasivam and coauthors recently published an SRM study on endosomal trafficking. Also there, impressive images depicted the colocalization of individual NPs with endosomal structures. Their findings are also of particular interest for the design of polyplex systems, as SRM revealed that NPs accumulated in the EE tubuli and remain there. [135] As briefly discussed in the *Introduction* section, the role of such unproductive NPs is still discussed; however, it is expected that they might critically affect the biocompatibility of a delivery system.

Unfortunately, also SRM bears its drawbacks. Indeed, the most controversial fact affects the labeling; similarly to all techniques depending on fluorescent labeling, it can always be doubted whether or not all relevant structures received the tag, and if free tag without target structure may be imaged. Especially when colocalization is used to evaluate images, this consideration becomes of utmost importance. Additionally, the labeling density or number of fluorophores is inversely correlated to the image acquisition time, *i.e.*, techniques aiming at real-time visualization usually employ very little, ideally single fluorophores per visualized structure. Studies presented here show larger structures with multiple labels, thus, again (similar as discussed for EM) only represent a frozen state of the dynamic process to be investigated. Nevertheless, the field is developing fast, and we expect further milestones to be presented in the coming years.

### 3.3 Discrepancies between different cell types

As introduced in Section 2.2, the exact mechanisms of intracellular trafficking can vary quite drastically among different cell types. It is thus not surprising that identical delivery vectors can perform significantly different depending on the cell type they are targeted at.

In a brief study, Zhang and colleagues investigated the relationship between cellular uptake and endosomal trafficking in HeLa and HUVECs cells using fluorescent nanodiamonds (FNDs). Cellular uptake experiments revealed an at least comparable, although not identical, uptake pattern. Afterwards, the authors studied the endosomal escape ability in both cell lines by analyzing colocalization with the endosomal compartment. Colocalization was quantified by confocal microscopy after treatment with endo-lysosomal calcein staining. Eight hours after the treatment, the amount of colocalized FNDs was determined to be less than 25% in HUVECs compared to almost double in HeLa cells. In fact, whereas no significant differences in endosomal escape 4 h and 8 h after treatment was observed in HeLa cells, colocalization was decreased dramatically in HUVEC cells. The authors discussed possible explanations for the observed differences in the occurrence of a protein corona, different uptake pathways, and the different origins of the cells. [136] Although these discussions remain to be investigated in detail, it highlights the importance of cellular considerations regarding the endosomal escape efficiency of drug delivery systems in general.

A very detailed comparison of endosomal escape efficiency and kinetics in both 3T3 fibroblasts and Renca kidney carcinoma cells was conducted by Rädler and colleagues. [70] They performed a combination of uptake and endosomal escape studies to provide information on the yield-time distribution and the cargo load per endosome. For their studies, the authors chose a colloidal mesoporous silica (CMS) nanoparticle to create a fluorogenic sensor coated with a lipid bilayer in the second step. A photosensitizer was encapsulated, creating reactive oxygen species which, upon light activation, induce membrane rupture. They designed a redox sensible fluorometric dye-quencher to quench the CMS NPs until endosomal rupture. In brief, if the cell is activated by light, the photosensitizer is released, and the quenching effect is removed. The lysis event was therefore directly quantified *via* confocal microscopy.

To check whether cell-specific differences might influence the lysis events, Rädler et al. first investigated the size distribution of endosomes with Alexafluor (AF) 488-labeled Dextran and roughly determined the load photosensitizer per endosome. The size of the endosomes in Renca cells showed a considerably more heterogenous distribution and, overall, tended towards larger endosomes. In contrast, 3T3 fibroblasts contained a higher load of NPs per endosome. A possible explanation was found in the longer lifetime of the Rab5 protein, which is primarily involved in endosomal fusion and consequently in endosomal size (see *Chapter 2* for details). Indeed, Rab5 is overexpressed in several carcinoma cells, such as lung carcinoma and Hela cells, highlighting the importance of this finding for applying NP systems in oncology, investigating optimal dosage regimen for cancer treatment. Also, the endosomal escape kinetics within both cell lines showed drastic differences in that regard; whereas the lysis in 3T3 cells occurs rapidly, release in Renca cells followed a steady, continuous increase. The authors developed a stochastic model to describe their findings on the endosomal escape kinetics. It was concluded that the lysis rate must be considered a cell-specific parameter based on both lipid and protein composition of the endosome and is decreased by a factor proportional to the endosomal size. [70]

Similar to Rädler and colleagues, Vermeulen *et al.* conducted a study investigating the impact of endosomal size and membrane leakiness on endosomal escape. [51] The authors thereby focused on the differences between HeLa and ARPE-19 retinal pigment epithelial cells. An eGFP transfection experiment using a JetPEI/pDNA polyplex formulation revealed, at first, better efficiency in HeLa cells. Confocal microscopy was then utilized to assess the number of endosomal escape events in each cell line. Indeed, HeLa cells showed a more pronounced endosomal escape than ARPE-19 cells. Controversially, the cellular uptake in ARPE-19 cells was much higher. To gain a deeper understanding of the parameters affecting the endosomal escape performance, the authors analyzed the number of NPs per, as well as pH and mobility of the endosome. It was shown that HeLa cells, interestingly, take up about three times fewer nanoparticles into a single endosome. Moreover, the pH inside the ARPE-19 endosomes was slightly more acidic when compared to HeLa cells, which seems contradicting as, according to the proton sponge hypothesis, a decreased pH should lead to further endosomal swelling and enhanced endosomal escape. Notably, no significant differences were observed in the endosomal mobility of the two cell lines. In the next step, the authors investigated the endosomal size measured by labeling the fluid phase of the endosomes with FITC-dextran. Indeed, they revealed a tendency towards bigger endosomes in ARPE-19 cells. Based on the endosomal size, they calculated the amount of polymer necessary for endosomal burst in both cell types and concluded that endosomal escape events are to happen more likely in HeLa cells based on the smaller endosomal size.

Nevertheless, in HeLa cells, escape events were observed only in ∼10% of the endosomes. The authors also continued to investigate the endosomal leakiness to gain further insight. Hence, a calcein release assay was employed, and to enhance the informative value of the study, the authors further included two additional cell lines, *i.e.*, lung carcinoma lines H1299 and A549. Interestingly, the endosomal leakiness was most pronounced in H1299 cells, potentially referring to low endosomal escape efficiency due to the hypothesis that osmotic pressure within the endosome cannot buildup, hence, the endosome cannot burst. Overall, the results suggested that the endosomal escape efficiency is indeed correlating with the endosomal size; however, under the boundary condition that membrane leakiness is low. [51] This finding is consistently supported by a variety of studies that will not be discussed in detail. [137,138]

## 4 Implications for the design of novel polymeric formulations

When starting to design a novel formulation, scientists are generally confronted with a few axioms that set the boundary conditions framing their work. Even assuming minimal restrictions, this general framework typically compromises a fixed disease target, the type of nucleic acid used for the therapeutic approach, and eventually a preferred administration route. Among the various additional determining factors as discussed in the *Introduction* section, scientists will at one point have to address endosomal escape of their polymeric formulation; this is where this review will discuss design principles, considerations, and potential alternative strategies that can help to tune vector towards efficient endosomal escape.

With the therapeutic target as well as the type of nucleic acid being given, one can distinguish two scenarios; either, the nucleic acid sequence can be delivered to the cytosol compartment, *e.g.*, as this is the case for mRNA- or siRNA-based therapeutics, or the sequence must be trafficked into the nucleus. The latter seems to be an additional hurdle compared to cytosolic delivery, as indeed, many studies designed their particle systems first to reach the cytosol and enter the nucleus afterward. However, when carefully designing the vector under the awareness of the intracellular trafficking mechanisms, other, eventually preferable pathways can also be considered. [139] In this context, Ross *et al.* have presented polyplexes that, instead of being optimized to escape the lysosomal pathway, were trafficked *via* the EE, MVB, and Golgi, through the endoplasmic reticulum, into the nucleus. To redirect their delivery system within the cellular machinery, the authors modified the polyplexes with histone H3-targeting peptides. For their investigations on the cellular pathway the authors added chloroquine as lysosomotropic reagent to the corresponding formulations. Curiously, those formulations which target histones showed decreased transfection efficiency upon chloroquine addition, whereas non-targeted PEI complexes showed the expected increase in the transfection efficiency. This led the authors to conclude that targeted versions of the PEI polyplexes follow a different path circumventing the lysosomal compartment. Indeed, by tracking the population of their polyplexes both along the envisioned pathway and along alternative routes through colocalization with the respective Rab-proteins involved, Ross *et al.* were able to demonstrate the success of their strategy impressively.

[140] In most cases, however, and especially when aiming to reach the cytosol, evasion of the endosomal escape bottleneck is not feasible. Therefore, the classical path through the endosomal compartment is the commonly observed route.

### 4.1 Poly- and micelleplexes to complex different types of nucleic acids

**Fig. 4:**
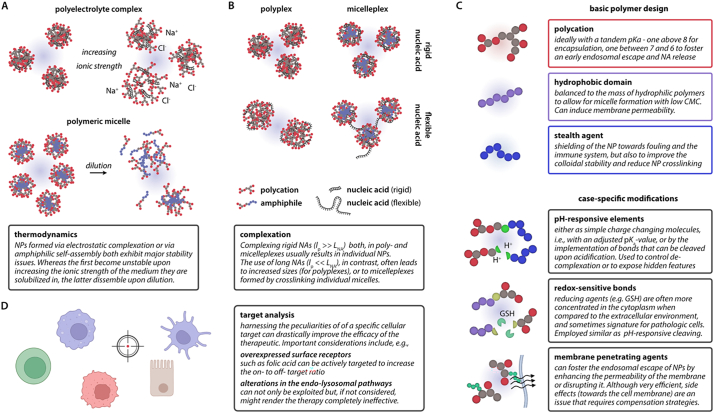
Toolbox for poly- and micelleplex design. In addition to the differentiation between electrostatic complexation and hydrophobic self-assembly (A), the physical properties of the nucleic acid sequence also influence the delivery system's structural properties (B). The three main polymeric building blocks comprise cationic, hydrophobic, and inert moieties. Additionally, pH- or redox-responsive and membrane penetrating agents might be incorporated in the formulation (C). To exploit the maximum specificity of a delivery system, the design should consider the peculiarities of the envisioned target (D).

At this point, considerations on whether to employ a poly- or micelleplex system become pressing (Fig. 4A). Thermodynamically, the properties of polyplexes are mainly governed by coulombic forces: in detail, the formation of an electrostatic complex is characterized by a reduction in the enthalpy *H* of the system accompanied by a loss in the systems’ entropy *S*. Overall, however, the Gibbs free energy *G* = *H* - T*S* is decreasing thus rendering the electrostatic complexation a thermodynamically favorable state [[141], [142], [143]]. Intuitively, an essential measure for all polyplex systems is the point at which the complex reaches overall charge neutrality, as in theory, this point reflects the thermodynamic equilibrium. For the nucleic acid cargo, it can be assumed that the phosphate group of every base carries one negative charge under all physiologically occurring conditions. Yet, for the polycationic counterpart, the actual charge depends on the protonation state and, therefore, on the p*K*_a_ value of the protonable groups present. Yu *et al.* suggested that for polyplexes comprising PEI, charge neutrality at physiological pH is reached at an N/P ratio of 3. Of course, this value can vary depending on the molecular structure of the PEI molecule, *i.e.*, the ratio of primary to secondary to tertiary amines, as the different amines become protonated at different pH levels also depending on their geometric accessibility. Formulations with N/P ratios above the threshold of charge neutrality can therefore be expected always to contain free/uncomplexed PEI. [144] Notably, free PEI is known to improve both cellular uptake and especially knock-down efficiency significantly. However, whether or not free PEI in a pharmaceutical formulation is desirable remains questionable. [145] As a secondary effect, it was observed, that drastically increasing the amount of polymer in a formulation also reduces the amount of nucleic acid molecules per complex – down to nucleic acid monomers [146]. In addition, the formation of polyplexes and their stability crucially depends the polymer architecture (see under 4.2) on the environmental conditions, in particular the ionic strength and dielectric constant of the solvent they are dispersed in [147]. Thus, it is not surprising that polyplexes have often been observed to dissociate or aggregate once exposed to biological fluids. [148]

Here, micelleplexes can offer distinct advantages. In fact, micelleplexes combine two driving forces that facilitate their colloidal stability, *i.e.*, coulombic and hydrophobic forces. [149] In contrast to coulombic complexation, hydrophobic self-assembly is almost exclusively driven by changes in the systems’ entropy. [150] In this context, an important measure for all amphiphiles/surfactants is the critical micelle concentration (CMC). The CMC reflects the point above which the loss in entropy resulting from clustering the amphiphiles is overcome by a gain in entropy due to the release of the solvation cages around the hydrophobic sites. [151] *Vice versa*, the kinetically trapped state of purely micellar structures is weakened upon dilution, and the micelles may disperse again. The micelleplex, as it utilizes both driving forces, can thus be considered significantly more stable. Coulombic forces can protect the micelleplex from disassembly at low polymer concentrations, whereas hydrophobic interactions are comparably resistant to the exposure of solutions comprising physiologically occurring mono- and divalent ions. [152,153] Obviously, the improved colloidal stability has its prize: although hydrophobic domains can also improve the cellular uptake and endosomal escape through intercalation with the plasma membrane, the same mechanism typically reduces their cytocompatibility. [154]

Regarding the considerations related to charge neutrality and free polymer, micelleplexes behave considerably differently from purely electrostatic complexes. Indeed, by adding entropic forces to the system, the range of thermodynamically stable N/P ratios becomes broader; therefore, overcharged systems can also exist. [155] A slightly overcharged system can be advantageous (see discussion in section 3.2.2). Notably, an excess in polymer excipient, especially when cationic, always enhances cytotoxicity. [156] The consequence is an optimization problem, *i.e.*, up to which point can the improved therapeutic efficacy and, therefore, the possibility to decrease the dosage compensate for the lower level of biocompatibility.

### 4.2 Characteristics of nucleic acids and polymers and their influence on complexation

At the same time, with the decision of the polymeric system, the type of nucleic acid comes into play. In fact, the physio-chemical characteristics of the nucleic acid - especially their length and whether they are single or double-stranded - play a crucial role in the complexation with the matrix polymer (Fig. 4B). [143,[157], [158], [159]] Whether the nucleic acid (NA) is single- or double-stranded critically influences its persistence length *l*_p_; for single-stranded nucleic acids such as ssDNA, pDNA, or mRNA, *l*_p_, depending on the environmental conditions, is usually in the range of a few nanometers. [160,161] The values for their double-stranded counterparts are approximately one order of magnitude greater. [162,163]. Connecting this information to the overall length *L*_NA_= N x *L*_bp_ of the nucleic acid (whereas one base pair (bp) has an average length of *L*_bp_ = 0.34 nm), three groups can be identified, flexible (*L*_NA_ >> *l*_p_), semiflexible (*L*_NA_ ≈ *l*_p_), and stiff (*L*_NA_ << *l*_p_), nucleic acids.

In 2019, Reineke and colleagues directly compared polyplexes with micelleplexes for the delivery of pDNA. [164] The authors employed the poly(2-(dimethylamino)ethyl methacrylate) (PDMAEMA) as a cationic block with similar molecular weight of all systems compared. PDMAEMA was used either as a homopolymer or coupled with poly(n-butyl methacrylate) (PnBMA) to form an amphiphilic AB-block. Additionally, both systems were synthesized with and without PEGylation, resulting in four polymeric systems. Polyplexes formed from pDNA with either PDMAEMA or PDMAEMA-PEG thereby morphologically significantly differed from their amphiphilic counterparts: whereas the first exhibit (partially large) spherical structures with the nucleic acid encapsulated in the core, the latter exhibit a bead-on-a-string geometry. Cryo-TEM imaging revealed that the relatively long pDNA acts as a crosslinker between individual micellar structures. Micelleplexes outperformed their purely hydrophilic counterparts dramatically, not only regarding cellular uptake but also in terms of transfection efficacy. To quantify the transfection efficiency eGFP positive cells were counted after the transfection with an eGFP encoding pDNA. At an N/P ratio of 10 only 2% of HeLa cells were found positive for eGFP expression, whereas 45% of Hela cells found positive when using the micelleplex counterpart for the transfection. The same pattern was observed in HEK 293T cells; in contrast to approximately 40% of the cells which were counted positive when using jetPEI, >80% of GFP positive cells were detected when employing micelleplex formulation [164].

Of note, variations in the size of the investigated complexes might have an effect on the preferred uptake pathways. Yet, the authors found that cellular uptake in all cases took place *via* the same caveola-mediated pathway. Consequently, the authors argued that the better performance regarding cellular uptake and endosomal escape might result from the micelleplexes being overcharged, *i.e.*, exhibiting a higher number of cationic groups bound to the complex. Interestingly, it was speculated about the role the nucleic acid conformation plays in transfection efficacy; The authors hypothesized that the condensed, folded structure of the pDNA in the polyplex might negatively affect accessibility and consequently impact transcription. [164]

Different authors around Reineke studied the complexation behavior of micellar systems in two additional studies; to do so, a similar system, *i.e.,* an amphiphilic PDMAEMA-PnBMA block co-polymer, was used. The scientists not only investigated the influence the length of the nucleic acid has but also how the N/P ratio affects the conformation of the assembly. Considering the two parameters, four scenarios can be identified: in the case of excess of polymer, long nucleic acid chains fostered the formation of bead-on-a-string structures, as discussed above. In contrast, short DNA strands lead to the formation of uniform, mostly individual micelles. Reducing the amount of polymer improves the individual structure formation when loading long DNA sequences, whereas aggregates are observed when micelles are complexed with short DNA. Additionally, when the amount of polymer was too low, the population was observed to become more heterogenous, which together with the presence of free nucleic acid prohibits those formulations from becoming pharmaceutically interesting. [165]. In the second study, the influence of a non-ionic corona was investigated in detail by employing a combination of cryo-TEM with different scattering techniques. The authors found that a non-ionic corona improves the colloidal stability of the micelleplexes when exposed to environments with varying ionic strength. Moreover, it was shown that the number of micelles per micelleplex decreases with increasing size of the PEG block attached to the PDMAEMA-PnBMA copolymer. [166]

In addition to the general type of polymer, and the characteristics of the nucleic acid sequence, also the detailed chemical structure of the used polymers can have a considerable impact on the complexation (Fig. 4C). [157] The chemical structure here comprises both stiffness of the polymer (which molecular weight and the degree of branching), as well as density and p*K*_a_ of the amine moieties. The chemical structure or architecture of the polymer, respectively, can critically determine the accessibility of charged groups and therefore the strength of electrostatic interactions with the nucleic acid. The role of the molecular weight seems to be understood quite well, and it is generally accepted that polymers below a certain weight threshold cannot efficiently complex nucleic acids. Correlations between degree of branching and the biological performance, however, can rather be related to the fact that the amount and type of amine group differs in branched polymers than to geometrical/spatial considerations. [[167], [168], [169], [170]]

The density and p*K*_a_ of the amine moieties was investigated by Kim *et al.* in two different studies. In both projects, the authors examined polyplexes formed from pDNA with either alpha- (APL), eta-polylysine (EPL), or a blend of both. Considering both complexation and cellular uptake efficiency, polyplexes formed from APL outperformed those formed by EPL, which agrees well with the fact that APL exhibits a higher p*K*_a_ value. In detail, the authors evaluated the transfection efficiency of their two systems in HepG2 and Hek293 cells. Interestingly, the transfection efficiency of APL/pDNA formulation in HepG2 cells remained equal when increasing the N/P ratios from 5 to 40. In contrast, the EPL/pDNA formulation in both cell lines displayed an improved transfection efficiency with increasing N/P ratios. Although the absolute transfection efficacy of APL/pDNA was superior in all samples, the single, rather high p*K*_a_ of APL was hypothesized to become an obstacle regarding decomplexation. [171] By blending the two polymers, APL and EPL, the authors were indeed able to find an optimum for their system; in terms of transfection efficiency this optimal balance between tight binding necessary to stably complex the cargo and loose binding to ensure its release was found at a minimum of 50% APL. Of note, the transfection was even improved further by incorporation of endosomolytic ATP. One, however, should be aware that complexes formed with high amounts of EPL exhibited considerably larger hydrodynamic radii when compared to their APL counterparts, which, in addition to the different p*K*_a_, might have impacted the uptake performance. [172]. In agreement with our discussion under Section 3.2 the ideal polycation/blend should exhibit a tandem p*K*_a_. Particularly, it is suggested to have one p*K*_a_ between 10 and 8 to enable efficient complexation and condensation of the nucleic acid and another one between 7.25 and 6.25 the facilitate endosomal escape and cargo release. [109]

Notably, even if similar components are being used to prepare a certain poly- or micelleplex formulation, the way those complexes are assembled can have further impact on their biological fate. Feldmann *et al.* in this context, found that one and the same formulation, when prepared *via* either bulk mixing vs. a microfluidic approach can exhibit significantly differing physical properties. These variations are critically reflected in the size of their micelleplexes which, as a consequence, influenced the uptake mechanisms and intracellular routes of the respective species [173].

### 4.3 Stimuli-responsive systems and induced destabilization of the endosomal membrane

Over the years, a multitude of delivery systems have been engineered to react as a response to certain triggers, and this similarly holds true also for poly-and micelleplexes. In many cases, stimuli-responsiveness of the carrier is utilized to either release the cargo in a certain environment or actively interact with the surroundings at a particular point along the delivery route. The advantages of such systems are obvious and include improved stability in off-target regions, as well as the possibility to specifically target diseased areas (Fig. 4C). [[174], [175], [176], [177]]

The most frequently explored trigger is undoubtedly a change in the pH value of the environment. In fact, many of the polyplex systems discussed above can be considered pH-responsive if their protonation increases upon acidification of the endosomal compartment. In addition to the beneficial effects of pH-dependent protonation on the endosomal escape, as discussed in Section 3.2, such changes in the polymer charge can be utilized to trigger additional effects. Yu *et al.* provided a strategy where the siRNA-loaded micelleplex releases Amphotericin B (AmB) when the pH level of the environment drops. In brief, *in vitro* studies were performed adding 1% (w/w) AmB to the formulation. This addition of AmB resulted in 80% Luciferase silencing in A549-Luc cells which corresponds to a 1.8-fold increase compared to control formulation. As AmB is known to mediate membrane pore formation, it was concluded that this strategy led to a significant improvement of the endosomal escape of the micelleplexes and, subsequently, the knockdown performance of the therapeutic siRNA. [178]

In the context of polymeric drug delivery systems, however, the term ‘pH-responsive’ is more commonly used for irreversible processes, such as the acidic cleavage of certain polymer structures. Cheng and coauthors, for example, proposed a pH-responsive micelleplex system to deliver pDNA. To allow for a pH-induced decomplexation of the micelleplex, the authors synthesized a pH-sensitive block copolymer comprised of a polycationic block and a pH-sensitive hydrophobic block. Upon acidification along the endo-lysosomal pathway, benzoic imines of the hydrophobic moieties are cleaved, thus destabilizing the complex and releasing the cargo. The highest luciferase expression and percentage of transfected cells was observed when transfecting HeLa cells with this pH-responsive formulation The performance of the system however notably decreased when trying to transfect different cell lines such as Z310 choroid plexus cells and primary neutral progenitor cells thus once more highlighting the relevance of the cellular model. Despite the cellular differences, the authors observed statistically relevant improvement when employing the SP formulation *in vivo*. [179] Another promising strategy was introduced by Cheng *et al*., who prepared micelleplexes, which shed their PEG layer in acidic environments. By dispersing the PEG layer, the cationic groups of their polymer become exposed. The authors hypothesized that this exposure of protonated amines is responsible for the improved endosomal escape *via* the proton sponge effect and, consequently, the knockdown efficacy. The same authors presented a polymeric system which they refer to as *virus-inspired polymer for endosomal release*, short *VIPER*. Also here, the protective layer of VIPER/pDNA complexes was dispersed upon acidification. Inspired by the trafficking mechanisms employed by adenoviruses, however, this shedding led to the exposure of membrane lytic melittin moieties of the VIPER polymer, thus facilitating endosomal escape [180]. To further allow for co-delivering the hydrophobic cytostatic camptothecin, Chen and colleagues, in addition to a pH-responsive shedding of a stealth layer, employed a redox-sensitive mechanism in their delivery system, thereby rendering their approach dual-stimuli responsive. Transfection studies revealed the ability of camphothericin to significantly improve the transfection efficiency in 4T1 breast cancer cells. The authors explain the positive influence of camphothericin with the destabilization of the polyplex through its hydrophobic moieties and therefore improved pDNA release [181].

Certainly, harnessing the presence of reducing substances to cleave pre-installed redox-sensitive bonds is the second most common approach to creating a stimuli-responsive system. Indeed, many reducing substances can be found intracellular, and they often exhibit significantly higher concentrations when compared to the extracellular environment. Furthermore, some reducing substances such as glutathione (GSH) or matrix metalloproteinase 2 (MMP-2) are additionally overexpressed in certain pathologic cells and can thus be used to improve the on- to off-target ratio. [177] Similarly, as introduced for pH-responsive systems, redox responsiveness can be employed to accelerate decomplexation and cargo release [[182], [183], [184]]. Wang *et al.* further improved their system by incorporating cell-penetrating peptides, which, upon the shedding of stealth layers after the cellular uptake, become exposed to facilitate endosomal escape [185]. The latter study highlights a critical problem with agents that actively destabilize or disrupt the endosomal membrane; those substances, if not shielded, would similarly impact the integrity of the cellular membrane; and even if strategies, as presented by Wang and colleagues, manage to circumvent this issue, the literature indicates that also excessive rupture of lysosomes is associated with apoptosis through the release of cathepsins and calcium ions. Therefore, it is crucial to additionally time the endosomal escape to the early stages in the endo-lysosomal pathway to limit the release of such proapoptotic molecules. [186,187]

An inherent problem of triggers such as pH, enzyme levels, or redox is generally low specificity. Consequently, their *in-vivo* performance often falls short of expectations. Recently, Kimna *et al.* presented an approach using specific nucleic acid sequences as a trigger. In brief, the authors crosslinked biopolymeric nanoparticles using specifically designed self-complementary DNA sequences. Those sequences were designed so that the crosslink can be opened up upon exposure to a trigger sequence. This trigger sequence, therefore, must exhibit a higher binding affinity to the crosslinking strand than to its identical counterpart. After separating the crosslinkers, the NP system disintegrates, thus releasing the cargo. By designing the DNA crosslinks to be responsive to certain signature sequences overexpressed in cancer cells (in this case, miRNA-21 in Hela cells), this strategy was not only shown to be cell-specific; in addition, a downregulation of the overexpressed genes can be expected when binding to the crosslinker strands. [188] Such an approach, up to now, has not been employed for poly- or micelleplex systems. It can be speculated, however, that combining such an approach with optimized polymeric compounds could initiate a new branch of disease-specific gene therapy.

### 4.4 Active modeling of intracellular trafficking

As discussed in the *Introduction* section, it is indisputably the goal of every therapeutic to achieve the maximum possible on- to off-target rate. To enable an efficient nanocarrier accumulation within particular tissues and/or cell types, *targeting* can be achieved by utilizing certain molecular interactions between the carrier and its target. [189]

Importantly, ligand-modified NPs which are preferably taken up by a certain cell types, not only improve the on-to off-target rate regarding the cellular uptake. Indeed, cargos taken up *via* certain receptors might be trafficked differently from the “*standard*” endo-lysosomal pathway. Few recent studies challenged the question of whether or not it is possible in the sense of rational drug design to harness a controlled targeting of a particular endosomal maturation state and consequently avoid clearing from cells in early stages (Fig. 4D).

To address this topic, we decided here to look at the concept of ligand-mediated targeting in dendritic cells (DCs). An example of the involvement of different endosomal pathways which could potentially be harnessed by receptor targeting can be found in the internalization pathway of C-type lectin receptors (CLRs) expressed on the surface of DCs. CLRs, as one group of pathogen-recognition receptors, can generally be divided into type I and type II receptors. [190] Type I CLRs include the MMR (CD206) and DEC-205 receptors. Type II CLRs include, among serval others, also the DC-SIGN (CD209) receptor. Whereas literature refers to the MMR (CD206) as a receptor for single mannose residues, DC-SIGN binds more effectively to N-linked high-mannose oligosaccharides and branched fucosylated structures [191]. MMR undergoes a continuous recycling process even without ligand-binding, as it is trafficked through the EE and recycled to the cellular surface. It is possible, therefore, that ligand-modified nanoparticles could be exocytosed during this recycling process if the ligand is not shed from the nanoparticle intracellularly. Through the recycling process, MMR potentially avoids endo-lysosomal fusion using a glycopeptidolipid-mediated inhibition mechanism [192,193]. In contrast, in immature DCs, DC-SIGN traffics the antigen after ligand binding directly into the LE or lysosomes. Therefore, DC-SIGN allows in immature DCs for ligand degradation before recycling of the receptor to the plasma membrane. In mature DCs, the receptor is recycled through the EE, which highlights the subtle differences in the receptor pathway depending on the maturation status of DCs and influences drug-carrier design in term of endosomal escape when targeting DC-SIGN in a certain maturation status of DCs. [190] Evidence for harnessing CD206 and CD209 targeting strategies resulting in different downstream effects was reported by Raviv and colleagues. The authors investigated formulations based on either mono- or tri-mannose modified PEG-bPEI. They chose CMVLuc and GFP plasmids for transfection and a DC2.4 cell line expressing both CD206 and CD209. The formulation functionalized with tri-mannose indeed showed a 3-fold increase in the transfection efficiency. Corrected on the single sugar residues, however, this improvement shrank to only ≈1,3-fold. The authors hypothesized that the orientation of mannose oligosaccharides might not fit the carbon recognition domains of CD209 and, therefore, did not lead to an enhanced effect. Notably, it was possible to significantly decrease the transfection efficiency by adding free mannose in order to inhibit nanoparticle-receptor interaction. [194] Unfortunately, not only the targeting itself seems to play a role in such ligand-mediated targeting strategies. Controversially, White *et al.* studied mono-mannosylated and tri-mannosylated liposome formulations. Although in monocyte-derived DCs (MoDCs) they observed an increased cellular uptake of tri-mannose functionalized particles when compared to those modified with mono-mannose, no enhanced DC activation was detected. [195]

As depicted above, targeting *via* receptors and their influence can be a challenging endeavor. In this context, we also had a closer look at folate receptor-mediated uptake. It is suggested that Folate receptor alpha (FRα) can be internalized *via* both clathrin-independent and clathrin-dependent pathways, depending on the concentration of FRα. An overexpression of FRα on the surface can, for example, be found in a range of epithelial tumor cells, including breast, lung, and ovarian cancer [196]. Therefore, targeting the folate receptor has become a promising strategy in cancer therapy and diagnostics.[197] The results of FR targeting are often impressive, and it is not surprising that a multitude of studies employed folic acids conjugate to improve the uptake performance of their delivery systems. [198]

Concerning efficient binding, Jones *et al.* observed a stronger binding of multivalent bound FA on the surface of functionalized PEI-PCL-PEG micelles to FRα when compared to free FA. In line, atomic force microscopy force measurements revealed a higher binding probability and binding avidity of folate decorated micelles. Uptake and knockdown experiments were performed using the FRα expressing ovarian cancer cell line SK-OV-3/Luc. The Luciferase knockdown efficiency was significantly higher using folate decorated micelleplexes when compared to non-decorated micelleplexes. However, it was not possible to observe a gene knockdown after applying receptor competition using a minimum of 1 mM free folic acid. Uptake experiments revealed a greater amount of micelleplexes to be located extracellularly over time. This suggested that some micelles were still bound to the receptor after the expected receptor recycling time of ≈5 hours [199]. Jones et al. also injected the respective targeted and non-targeted micelleplexes into an ovarian cancer mouse model leading to a knockdown of 62% after 48 h and the end of the transient knockdown being observed after 72 h. Unfortunately*, in vivo*, only a slight benefit of the targeted vs. non-targeted formulation was observed 24 h post-administration with successful uptake in metastases, however [200].

Improved knockdown efficiency seen in *in vitro* models is likely to be associated with the alterations in intracellular trafficking after folate mediated uptake. Furthermore, cellular differences were also reported; whereas in certain cell types, such as CHO and Cos-7 cells, folate mediated uptake results in trafficking into the endocytic recycling compartment, in other cell types, cargos are trafficked efficiently along the endo-lysosomal pathway [196]. Additionally, it was reported that FA receptor-containing endosomes might become less acidic when compared to their receptor-free counterparts. [201] In this context, a study conducted by Gary *et al.* seems particularly interesting; already 10 years ago, the authors investigated the knockdown performance of polyplexes, micelleplexes, as well as folate-conjugated micelleplexes. The results showed that micelleplexes outperformed the polyplex system regarding GAPDH silencing despite their comparatively low uptake performance. Yet, the role of folate-conjugation became certainly puzzling. Although the uptake of micelleplexes *via* the folate-receptor was considerably higher when compared to their unmodified counterparts, the overall knockdown efficacy remained on a similar level. The authors concluded that apparently, uptake of the nanocarriers was not the rate-limiting factor. [202]

However, one could similarly hypothesize that the folate-mediated uptake pathway resulted in an alternation of the intracellular route less favorable for the endosomal escape, *e.g.*, via the recycling compartment. Certainly, we can conclude that also targeting routes must be seen in context with the particular delivery system. One should certainly remain aware that ligand-mediated targeting can similarly lead to favorable or unfavorable trafficking routes. A separate investigation of uptake and knockdown performance is thus imperative for the design of polymeric vectors. Additionally, the presence of motives on the surface of the nanoparticle that is intended to bind to target structures specifically might similarly counter shielding strategies; In fact, it has to be weighed off carefully if the advantage of ligand-mediated targeting covers up the potential loss of efficacy caused by increased aggregation, proteolytic degradation, or renal elimination. [203]

## 5 Conclusions & Outlook

Even though primarily focusing on the intracellular trafficking of poly- and micelleplexes in this article, it became evident that designing such delivery systems is everything but a straight one-way road. Indeed, a multitude of reflections is required to find an optimal balance between the multitude of parameters affecting the performance of the vector. In general, however, *in vivo* studies taught us one rule: simple and robust systems are always preferred compared to overengineered vectors that are sensitive towards the slightest variations in environmental conditions. Similarly, this ‘*simplicity-rule*’ holds when translating the system into the clinic and, at the latest, when commercial aspects come into play [204].

Two elements will become crucial for future developments: the consequent unveiling of the remaining biological details of endosomal processing bears time- and cost-efficient strategies to screen formulations regarding their efficacy rather than employing polymer libraries. A thorough understanding of the underlying biological principles could ultimately result in the capability to modify or manipulate the intracellular machinery actively. Despite still being in their infancy, first approaches have, for example, demonstrated how specific substances, such as adamantane-like compounds, reduce the expression of Rab7 and thus delay intracellular trafficking of endosomal contents. Despite the rather narrow window between effective and toxic doses for these compounds, we envision manifold possibilities for the maturation of this approach. [205] The latter, *i.e.*, the optimization of screening strategies, is likely supported by the ongoing development of machine- and especially deep-learning methods. Together with sophisticated simulation approaches, first strategies are emerging to pre-design polymer variants regarding structural parameters such as charge density, p*K*_a_, molecular weight, or branching *in situ*. This preselection will subsequently be tested with the support of traditional wet lab methods and, if necessary, optimized iteratively. [[206], [207], [208]]

In addition, also wet lab techniques might benefit from novel approaches; as understanding endosomal escape mechanisms is critically challenged by experimental boundary conditions that are hard to control and maintain, sophisticated model systems could help push the current knowledge boundaries. In this context, membrane model systems have shown encouraging results to investigate the interactions with different polymer vectors, which could be correlated to their *in vitro* properties [209]. Furthermore, the use of isolated or artificial and/or isolated endosomes to study either specific pathways or parameter relationships under controllable boundary conditions [210,211] could be a promising strategy to verify theoretical investigations of the endosomal physics as discussed in Section 3.2.2.

Biological compatibility and immunogenicity remain omnipresent issues despite all those technical aspects affecting therapeutic performance. The search for PEG alternatives as stealth agents is an ongoing journey, and different candidates, *e.g.*, polysarcosine [212], poly(carboxybetaine) [213], or poly(methacrylamide) [214], have been proposed that might in future replace the current gold standard. Fast progress can be expected in this field, as the topics of immune shielding, as well as improving colloidal stability and cytocompatibility, are equally essential affairs for many other nanoparticulate systems, including the currently used lipid-based covid-19 vaccines. Correspondingly, inventions can be seen on the polymer side: just recently, Lee *et al.* presented DNA-inspired polycations as drug delivery systems for RNAi therapy, which, especially regarding biocompatibility, outperformed not only PEI and PLL but also lipid NPs [215]. In this context, most experts in the field agree to replace the remaining non-degradable polymers with their respective degradable counterparts. The key challenge thereby remains to maintain the high efficacy of established systems using non-degradable materials.

## Declaration of Competing Interest

The authors declare no competing interest.

## Data Availability

Data will be made available on request.
